# A prospective longitudinal study of performance status, an inflammation-based score (GPS) and survival in patients with inoperable non-small-cell lung cancer

**DOI:** 10.1038/sj.bjc.6602591

**Published:** 2005-05-03

**Authors:** L M Forrest, D C McMillan, C S McArdle, W J Angerson, K Dagg, H R Scott

**Affiliations:** 1 University Department of Surgery, Royal Infirmary, Glasgow G31 2ER, UK; 2 Department of Respiratory Medicine, Wishaw General Hospital, Lanarkshire ML2 0DP, UK

**Keywords:** longitudinal study, non-small-cell lung cancer, ECOG-ps, GPS, survival

## Abstract

The value of an inflammation-based prognostic score (Glasgow Prognostic score, GPS) was compared with performance status (ECOG-ps) in a longitudinal study of patients (*n*=101) with inoperable non-small-cell lung cancer (NSCLC). At diagnosis, stratified for treatment, only the GPS (HR 2.32, 95% CI 1.52–3.54, *P*<0.001) was a significant predictor of survival. In contrast, neither the GPS nor ECOG-ps measured at 3–6 months follow-up were significant predictors of residual survival. This study confirms the prognostic value of the GPS, at diagnosis, in patients with inoperable NSCLC. However, the role of the GPS and ECOG-ps during follow-up has not been established.

Non-small-cell lung cancer (NSCLC) is the most common cause of cancer-related death in North America and Western Europe. Most patients present with advanced inoperable disease and the majority die within 12 months. Disease progression is associated with a decline in both nutritional and functional status.

There is increasing evidence that the progressive decline of patients with advanced cancer is, at least in part, secondary to the presence of a systemic inflammatory response (Kotler, 2000; Scott *et al*, 2002). For example, it has been shown that the presence of a systemic inflammatory response, as evidenced by an elevated circulating concentration of C-reactive protein and reduced concentrations of albumin, is associated with poor survival independent of stage and performance status in a variety of common solid tumours (O'Gorman *et al*, 2000; Scott *et al*, 2002). Specifically, we have shown that a combination of C-reactive protein and albumin, the Glasgow Prognostic score (GPS), has prognostic value, independent of performance status, in patients with inoperable NSCLC (Forrest *et al*, 2003; Forrest *et al*, 2004).

In the above studies, the GPS was measured at diagnosis. To date the value of the GPS as a method of monitoring of patients with inoperable NSCLC has not been assessed. The aim of the present study was, therefore, to examine, in a longitudinal study, the prognostic value of GPS and performance status in patients with inoperable NSCLC.

## PATIENTS AND METHODS

### Study design

Patients presenting with inoperable NSCLC (stages III and IV) to a multidisciplinary clinic at Wishaw General Hospital, Lanarkshire between January 2002 and December 2003 were studied prospectively. All patients had cytologically or histologically confirmed disease and were staged on the basis of clinical findings, chest X-ray and, where appropriate, bronchoscopy, liver ultrasound, isotope bone scan and computerised tomography of the thorax, according to the American Thoracic Society TNM classification (Mountain, 1991).

Weight, clinical stage and performance status (Eastern Cooperative Oncology Group, ECOG-ps) were recorded at the time of diagnosis. Performance status was assessed by a respiratory physician (KD, HRS). A blood sample was also obtained, at diagnosis, for measurement of white cell count, haemoglobin, albumin and C-reactive protein concentrations. These measurements were repeated 3–6 months after diagnosis.

Following diagnosis, patients were considered to have undergone active treatment if they received chemotherapy (mainly cisplatin based) and/or radical radiotherapy. Patients receiving palliative radiotherapy and/or palliative care (symptom control) were considered to have had supportive treatment.

The study was approved by the Research Ethics Committee at Wishaw General Hospital, Lanarkshire.

### Methods

Blood parameters, routine laboratory measurements of haemoglobin, white cell count, albumin and C-reactive protein concentrations were carried out. The coefficient of variation for these methods, over the range of measurement, was less than 10% as established by routine quality control procedures.

The GPS was constructed as previously described (Forrest *et al*, 2003). Briefly, patients with both an elevated C-reactive protein (>10 mg l^−1^) and hypoalbuminaemia (<35 g l^−1^) were allocated a score of 2. Patients in whom only one of these biochemical abnormalities was present were allocated a score of 1. Patients in whom neither of these abnormalities was present were allocated a score of 0.

### Statistics

Univariate survival analysis was performed using the Kaplan–Meier method with the log-rank test. Multivariate survival analysis and calculation of hazard ratios (HR) were performed using the Cox regression analysis. Deaths up to 30 November 2004 were included in the analysis. Where appropriate, comparisons of data from different time periods were carried out using the sign test. Analysis was performed using SPSS software (SPSS Inc., Chicago, IL, USA).

## RESULTS

The clinical characteristics of patients with inoperable NSCLC at diagnosis are shown in Table 1. The majority were male, over the age of 60 years and had ECOG-ps 0–1. Approximately half of the patients had stage IV disease. A total of 68% patients had an elevated C-reactive protein concentration; 10 patients had a low albumin concentration. The majority of patients (69%) had an abnormal GPS. In all, 42% of patients received active treatment.

During follow-up, 29 of 42 patients receiving active treatment died. Of 59 patients, 55 receiving palliative treatment died. At diagnosis, stratified for treatment, only the GPS (HR 2.32, 95% CI 1.52–3.54, *P*<0.001) was significantly associated with survival.

At the 3–6 months, 17 of the 42 patients who received active treatment died and further seven patients were unfit to attend the clinic, leaving 18 (43%) patients available for assessment. Of the 59 patients who received palliative treatment, 37 died and a further two patients were unfit to attend the clinic, leaving 20 (34%) patients available for the 3–6 month assessment (Table 2).

At the follow-up assessment (3–6 months), there was a reduction in ECOG-ps (*P*<0.01) and the GPS (*P*<0.10). At this time, stratified for treatment, neither ECOG-ps (*P*=0.082) nor GPS (*P*=0.139) were significantly associated with subsequent survival.

## DISCUSSION

The present study examined the longitudinal changes in an inflammation-based prognostic score (GPS) and performance status (ECOG-ps) in patients with inoperable NSCLC. Of the initial 101 patients studied, only 38 were available for the follow-up assessment. This high attrition rate is consistent with previous longitudinal studies of advanced cancer patients (McMillan *et al*, 1999; Vigano *et al*, 2000; Lundholm *et al*, 2004).

In the present study, at diagnosis, the GPS was superior to ECOG-ps in predicting survival. Therefore, these results are consistent with our previous cross-sectional studies of ECOG-ps and the GPS (Forrest *et al*, 2003; Forrest *et al*, 2004).

In contrast, at the 3–6 month assessment, neither ECOG-ps nor the GPS predicted survival independent of treatment. This probably reflects the small numbers of patients studied at this time. Also, the loss of prognostic value of ECOG-ps and the GPS at this time may be related to the time-dependent nature of these variables. For example, Vigano *et al* (2000) reported that, in terminally-ill patients with advanced cancer, the predictive value of both ECOG-ps and albumin concentrations decreased over time.

Prediction of survival in patients with common solid tumours remains problematical despite the sophisticated mathematical analyses and the many variables that have been analysed (Buccheri and Ferrigno, 2004). Currently, stage and performance status are the most commonly used routinely to assess likely outcome. The present and previous studies of the GPS highlights the independent value of the systemic inflammatory response as a prognostic factor in inoperable NSCLC. This may be in part because the assessment of stage and performance status reflects patient status at a specific point in time. In contrast, the GPS, based as it is on hypoalbuminaemia and the presence of an ongoing systemic inflammatory response, not only reflects current nutritional status but also predicts continuing nutritional decline of the patient (McMillan *et al*, 1998; O'Gorman *et al*, 1999; McMillan *et al*, 2001).

The GPS may also have a prognostic role in patients with other inoperable cancers. If this were to be the case, it would confirm the general importance of the systemic inflammatory response and suggest that the GPS may be useful as a framework to identify other inflammation-derived symptoms in advanced cancer patients. Finally, the GPS may be combined with other established prognostic factors to improve the prediction of survival in the patient with advanced cancer.

In summary, at diagnosis, the GPS is superior to ECOG-ps in predicting survival, independent of treatment received, in patients with inoperable NSCLC. As such, it offers the potential to stratify patients at diagnosis and who are being considered for active treatment. However, the role of the GPS or ECOG-ps during routine follow-up has not been established.

## Figures and Tables

**Table 1:** Diagnosis characteristics and survival of patients with inoperable NSCLC

	**Patients**	**Survival (months)**	
	**101**	**Median (95% CI)**	***P*-value**
*Age (years)*			
<60	18	11.9 (0.6–23.2)	
⩾60	83	7.9 (6.3–9.6)	0.458

*Sex*			
Male	62	8.6 (5.4–11.8)	
Female	39	7.4 (5.0–9.8)	0.344

*Stage*			
III	49	10.0 (7.2–12.8)	
IV	52	6.2 (4.6–7.7)	0.067

*Haemoglobin (g / l)*			
⩾12	67	8.4 (6.3–10.4)	
<12	33	8.2 (2.5–14.0)	0.724

*White cell count*			
⩽11 × 10^9^ / l	76	8.4 (6.4–10.4)	
>11 × 10^9^ / l	24	4.5 (0.0–10.4)	0.226

*C-reactive protein (mg / l*)			
⩽10	33	11.6 (3.3–20.0)	
>10	68	7.3 (5.2–9.3)	0.006

*Albumin (g / l)*			
⩾35	91	8.7 (6.9–10.5)	
<35	10	1.2 (0.0–2.8)	<0.001

*ECOG-ps*			
0	17	14.8 (9.5–20.2)	
1	44	6.5 (4.0–9.0)	
2	30	8.4 (6.5–10.3)	
3	10	1.2 (0.1–2.3)	0.003

*GPS*			
0	32	11.6 (4.2–19.0)	
1	59	8.4 (7.0–9.8)	
2	10	1.2 (0–2.8)	<0.001

*Treatment*			
Active	42	15.5 (10.7–20.4)	
Palliative	59	5.8 (3.7–7.9)	<0.001

GPS=Glasgow Prognostic score; 95% CI=95% confidence interval; ECOG-ps=Eastern Co-operative Oncology Group.

**Table 2:** Parameters of patients with inoperable NSCLC at diagnosis and 3–6 months later

	**At diagnosis**	**3–6 months later**	
	**(*n*=38)**	**(*n*=38)**	***P*-value**
*Age (years)*			
<60	8 (21)		
⩾60	30 (79)		

*Sex*			
Male	27 (71)		
Female	11 (29)		

*Stage*			
III	26 (68)		
IV	12 (32)		

*Haemoglobin (g / l)*			
⩾12	21 (55)	20 (16)	
<12	17 (45)	16 (42)	0.739

*White cell count*			
⩽11 × 10^9^ / l	29 (76)	26 (68)	
>11 × 10^9^ / l	9 (24)	10 (26)	0.705

*ECOG-ps*			
0	10 (26)	7 (18)	
1	14 (37)	10 (26)	
2	11 (29)	13 (34)	
3	3 (8)	8 (21)	0.005

*GPS*			
0	14 (37)	11 (29)	
1	22 (58)	15 (40)	
2	2 (5)	9 (24)	0.059

GPS=Glasgow Prognostic score; ECOG-ps=Eastern Co-operative Oncology Group.
